# Exploring aromatic components differences and composition regularity of 5 kinds of these 4 aroma types *Phoenix Dancong* tea based on GC–MS

**DOI:** 10.1038/s41598-024-53307-6

**Published:** 2024-02-01

**Authors:** Xiao-Ting Zheng, Xing-Yao Zeng, Xiao-Ling Lin, Dan-Sheng Chen, Yun Li, Jian-Jian Huang, Zheng-Chao Yu, Hui Zhu

**Affiliations:** School of Life Sciences and Food Engineering, Hanshan Normal University, Chaozhou, 521041 People’s Republic of China

**Keywords:** Gas chromatography, Liquid chromatography, Solid-phase microextraction

## Abstract

Different aromatic components do indeed give different tea flavors. There is still little research on whether there is a certain regularity in the combination and content of aromatic components in different aroma types of *Phoenix Dancong* (PDC) tea. This potential regularity may be a key factor in unraveling the relationship between reproduction and evolution in PDC tea. Here, the 5 kinds of these 4 aroma types PDC tea (Zhuye, Tuofu, Jianghuaxiang, Juduo, Yashixiang) were used as research materials in this study, the headspace solid-phase microextraction combined with gas chromatography–mass spectrometry was used to analyze the aromatic components of these PDC teas. The results showed a total of 36 aromatic components identified in this study. When conducting cluster analysis, it was found that similarity degree arrangement sequence of 5 PDC teas was Juduo, Tuofu, Yashixiang, Zhuye and Jianghuaxiang. Among these aromatic components, the 7,9-Di-tert-butyl-1-oxaspiro(4,5)deca-6,9-diene-2,8-dione, the 2-Cyclopenten-1-one, 3-methyl-2-(2-pentenyl)-,(Z)-, the 2,4-Di-tert-butylphenol, the 3,7-dimethyl-1,5,7-Octatrien-3-ol, and the 2-Furanmethanol,5-ethenyltetrahydro-.alpha.,.alpha.,5-trimethyl-,cis- are common to 5 PDC teas. This study aims to elucidate the similarities in the aromatic components of 5 PDC teas, revealing the major aroma-endowed substances of various aroma, and providing theoretical reference for further exploring the relationship between aroma type discrimination, variety selection, and evolution of PDC teas.

## Introduction

*Phoenix Dancong* (PDC) tea is a traditional and famous tea with a history of more than 900 years in China. PDC tea is an excellent monotypic selection from the Fenghuang Narcissus species, each monotypic plant having its own characteristics in form and flavor, and forming a variety. The tea that is harvested and made separately from these individual tea plants is called Dancong tea. PDC tea belongs to the oolong tea category and is a semi-fermented tea^1^. The finished product is dark brown with a golden, bright soup color, elegant aroma and unique flavor. Its quality is renowned both domestically and internationally. PDC tea is famous for its aroma type. According to traditional sensory evaluation, the finished tea can be categorized into Rougui Xiang, Zilan Xiang, Huangzhi Xiang, Xinren Xiang, Milan Xiang, Yulan Xiang, Jianghua Xiang, Guihua Xiang, etc.^2^. However, there is such an asymmetric phenomenon, the continuous development of tea cultivation technology, so that in the same aromatic tea and evolved a variety of varieties of tea, but people have a strong subjective awareness of the aroma characteristics of different types of PDC tea and lack scientific and effective unified standards. This leads to an incorrect assessment of a certain variety of tea as belonging to that type of aromatic tea.

Aroma is an important characteristic of tea quality^3^, and the essential difference between different aroma types of tea lies in the difference in the composition and content of their tea aromatic components^2^. Currently, several studies have analyzed the aromatic composition of PDC teas and concluded that the dominant flavors of tea due to the different contents and combinations of various volatile flavor compounds within the tea^4^. In the studies of tea, it has been found that although volatile compounds only account for a very small percentage of the total dry weight (around 0.01%), they have a significant impact on the flavor of tea^5,6^. In recent years, research on key aromatic compounds in tea has attracted much attention^7–10^. However, due to the relatively low content and complex types of aromatic compounds in tea, there have been few reports on the composition of aromatic components in different aroma types of PDC teas. There is even less research on the similarity or correlation between different types of PDC tea and different varieties of tea with the same aroma, which poses challenges to the development, selection of varieties, and study of evolutionary relationships in PDC tea. At present, the aromatic chemical species in aromatic components are commonly obtained by headspace solid-phase microextraction (HS-SPME) method^6,11,12^ and gas chromatography-mass spectrometry (GC–MS) is used to analyze and identify the various aromatic compounds in tea^13–15^. Due to the complexity and diversity of tea aromatic components identified by GC–MS, corresponding methods must be combined to analyze and interpret them, such as principal component analysis (PCA) and hierarchical clustering analysis (HCA) methods^16,17^, which are used to evaluate various tea varieties and are an important tool for determining the quality of tea and the differences in aromatic components across regions^18–20^. Therefore, focusing on the purpose of this study, these analytical methods can be used to uncover the key aromatic substances between different tea varieties, and to study the similarity or correlation between different aroma types of tea and the same aroma type with different varieties tea.

Previous studies have evaluated the aromatic components of different aroma types of PDC tea^2,4,21^, but the studies were not specific to that variety and key aromatic components. In addition, there is a lack of corresponding research reports on the systematic comparison and classification of aromatic components in different aroma types of PDC tea, which is not conducive to studying the differences between PDC tea varieties and identifying those that belong to the corresponding aroma types of PDC teas. Therefore, in this study, the HS-SPME combined with GC–MS was used to extract and identify the aromatic components of 5 PDC teas of these 4 aroma types, PCA and hierarchical cluster analysis methods were used in combination with visualization using Venn diagrams to present the similarities and differences of the aromatic components of 5 PDC teas. Together, these were used to reveal the typical aroma features and core aroma-enhancing substances, as well as the combination and content patterns of different aroma types of PDC tea. The results show the similarities and differences of the aromatic components of 5 PDC teas, revealing their typical aroma characteristics, the core aroma-endowed substances, and the regularity of the combination and contents of the aromatic components of the different aroma types of PDC teas, thus providing a scientific basis for the development of PDC tea, the identification of aroma types, the selection and breeding of varieties, and the study of evolutionary relations.

## Results and discussion

### Identification and relative content analysis of aromatic components in five kinds of PDC tea

**Figure 1:**
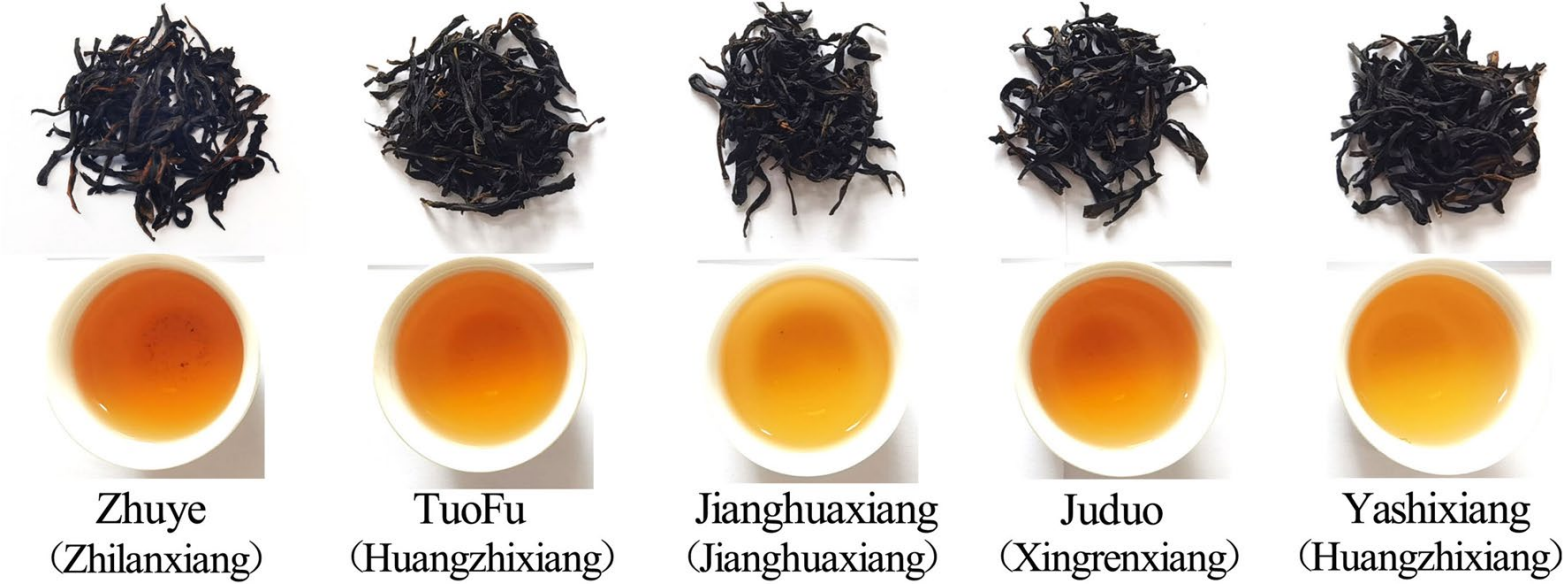
Finished tea after refining of 5 kinds of these 4 aroma types tea leaves, the corresponding brewed tea broths and the aroma type of tea to which they belong.

**Figure 2:**
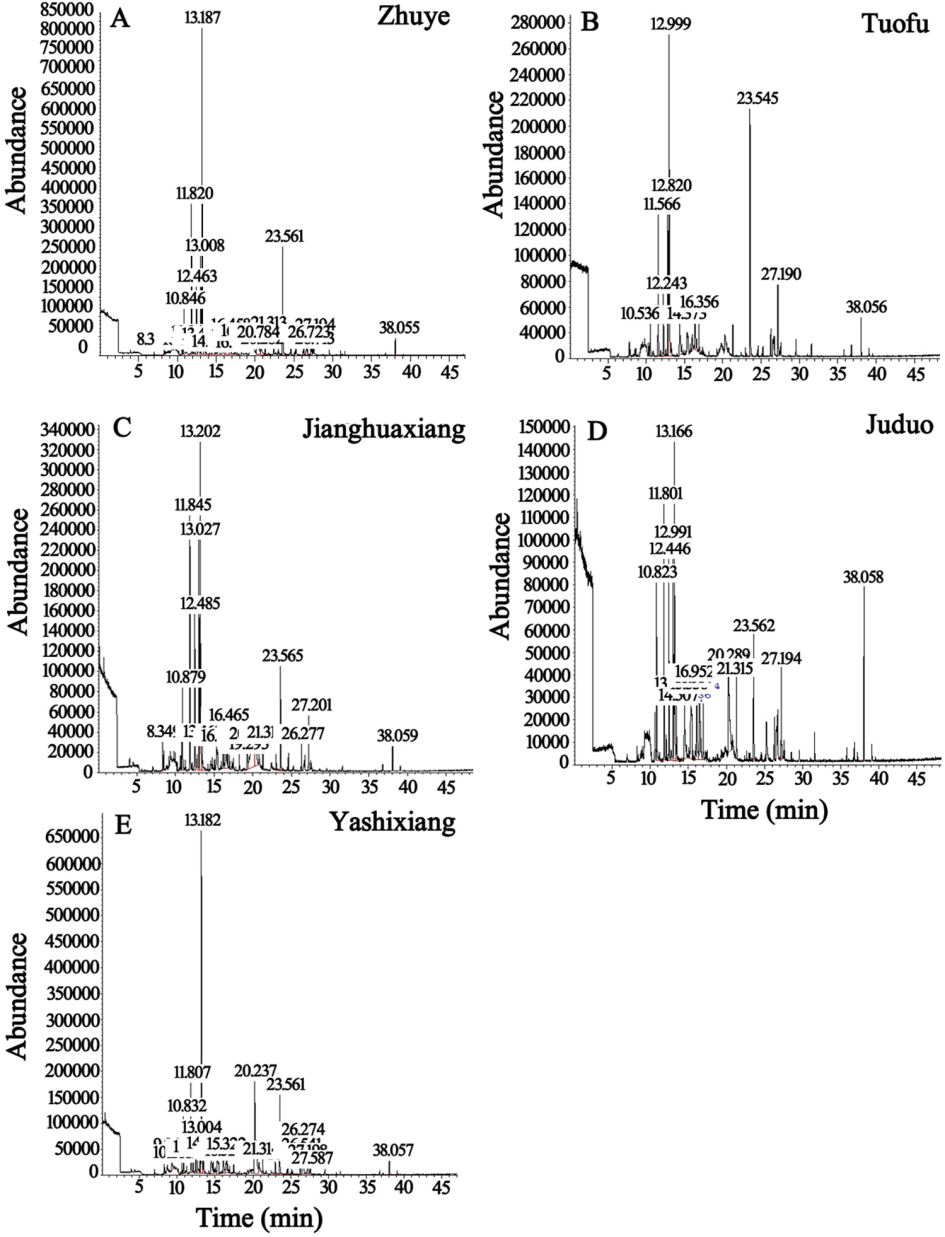
TICs of 5 kinds of these 4 aroma types PDC tea samples by gas chromatography-mass spectrometry (GC–MS) (**A**, Zhuye; **B**, Tuofu; **C**, Jianghuaxiang; **D**, Juduo; **E**, Yashixiang).

In this study, the teas of 5 kinds of these 4 aroma types PDC tea (Zhuye, Tuofu, Jianghuaxiang, Juduo, Yashixiang) were randomly selected (Fig. 1). The aromatic components of these 5 kinds teas were analyzed and identified using HS-SPME combined with GC–MS (Fig. 2), and the relative contents of each component were calculated using peak area normalization (Table 1 and Table S1). A total of 36 aromatic components were identified based on mass spectrometry data, relative retention time, and peak area. Zhuye identified a total of 19 aromatic components (Table 1 and Fig. 4B), including 4 alcohols, 4 ketones, 3 aldehydes, 2 phenols, 2 heterocycles, 1 lactones, 2 siloxanes, and 1 nitrogenous, which the relative content of the 3,7-dimethyl-1,5,7-Octatrien-3-ol had the highest relative content of 27.7%, followed by the 2-Furanmethanol,5-ethenyltetrahydro-.alpha.,.alpha.,5-trimethyl-,cis-(10.6%); Tuofu identified a total of 10 aromatic components (Table 1 and Fig. 4B), including 2 ketones, 2 phenols, 4 alcohols, and 2 lactones, which the relative content of the 3,7-dimethyl-1,5,7-Octatrien-3-ol reached the highest of 30.60%, followed by the 2-Cyclopenten-1-one,3-methyl-2-(2-pentenyl)-,(Z)-reached 18.06%; Jianghuaxiang identified a total of 16 aromatic components (Table 1 and Fig. 4B), including 3 ketones, 2 phenols, 5 alcohols, 2 heterocycles, 3 nitrogenous, and 1 aldehydes, which the relative content of the 3,7-dimethyl-1,5,7-Octatrien-3-ol had the highest relative content of 19.9%, followed by the 4-Hexen-1-ol,5-methyl-2-(1-methylethenyl)-, the 2-Furanmethanol,5-ethenyltetrahydro-.alpha.,.alpha.,5-trimethyl-,cis- and the Linalool relative contents were 12.7%, 12.1% and 11.7%, respectively; Juduo identified a total of 15 aromatic components (Table 1 and Fig. 4B), including 2 ketones, 1 phenols, 3 alcohols, 3 lactones, 1 heterocycles, 1 siloxanes, 3 nitrogenous and 1 aldehydes, which the 3,7-dimethyl-1,5,7-Octatrien-3-ol had the highest relative content of 14.8%, followed by the Indole with a relative content of 10.8%; Yashixiang identified a total of 13 aromatic components (Table 1 and Fig. 4B), including 4 ketones, 1 phenols, 2 alcohols, 2 lactones, 2 heterocycles, 1 siloxanes, and 1 nitrogenous, which the relative content of the 3,7-Dimethyl-1,5,7-Octatrien-3-ol had the highest relative content of 22.8%, followed by the Indole and the Bicyclo[3.1.1]heptan-3-one,2-hydroxy-2,6,6-trimethyl- with relative contents of 18.9% and 13.1%, respectively. Different tea varieties should have different aroma partitioning and composition characteristics^4,9,10^, and our results also support this conclusion (Fig. 2 and Table 1). However, we found significant differences in the aromatic components between different PDC tea varieties with the same aroma type. For example, in this study, Tuofu and Yashixiang, both of which originally belonged to the Huangzhixiang type of PDC tea (Fig. 1), showed significant differences in partitioning of aroma (Table 1). Therefore, we have to present the similarities and differences of the aromatic components of 5 PDC teas through the corresponding analytical methods, and find the composition and modulations of the aromatic components hidden in the tea samples.

**Table 1:** Relative content of each aroma component in 5 kinds of these 4 aroma types PDC tea samples.

Component	RT	Relative content (%)					Category
		ZY	TF	JHX	JD	YSX	
7,9-Di-tert-butyl-1-oxaspiro(4,5)deca-6,9-diene-2,8-dione	38.05	1.15 ± 0.10	2.41 ± 0.08	1.05 ± 0.03	4.63 ± 0.48	3.62 ± 0.10	Ketones
2-Cyclopenten-1-one, 3-methyl-2-(2-pentenyl)-, (Z)-	23.54	8.76 ± 0.36	18.1 ± 0.67	6.44 ± 0.24	4.67 ± 0.54	17.1 ± 0.43	Ketones
2,4-Di-tert-butylphenol	27.19	2.18 ± 0.15	5.39 ± 0.43	2.78 ± 0.12	3.39 ± 0.42	2.58 ± 0.03	Phenols
3,7-dimethyl-1,5,7-Octatrien-3-ol	12.99	27.7 ± 0.23	30.6 ± 2.75	19.9 ± 0.18	14.8 ± 0.91	22.8 ± 0.07	Alcohols
2-Furanmethanol, 5-ethenyltetrahydro-.alpha.,.alpha.,5-trimethyl-, cis-	11.56	10.6 ± 0.12	10.5 ± 0.77	12.1 ± 0.18	8.34 ± 0.45	4.00 ± 0.28	Alcohols
Linalool	12.82	8.09 ± 0.23	12.7 ± 0.15	11.7 ± 0.17	8.78 ± 0.58	–	Alcohols
Benzyl nitrile	14.37	3.19 ± 0.10	5.88 ± 0.27	–	4.61 ± 0.53	4.29 ± 0.41	Lactones
trans-Linalool oxide (furanoid)	12.48	5.57 ± 0.08	–	7.72 ± 0.26	–	2.08 ± 0.26	Heterocycles
6-Methyl-5-hepten-2-one	8.03	0.95 ± 0.06	–	2.39 ± 0.14	–	–	Ketones
Indole	20.29	5.88 ± 0.17	–	–	10.8 ± 0.45	18.9 ± 0.40	Heterocycles
3-Buten-2-one, 4-(2,2,6-trimethyl-7-oxabicyclo[4.1.0]hept-1-yl)-	26.27	2.04 ± 0.08	–	–	–	2.03 ± 0.01	Ketones
Terpineol	16.46	5.81 ± 0.09	–	6.07 ± 0.27	–	–	Alcohols
Cyclopentasiloxane, decamethyl-	15.33	3.97 ± 0.08	–	–	7.52 ± 0.68	4.91 ± 0.54	Siloxanes
Phenol, 4-amino-2-methyl-	10.53	–	3.12 ± 0.32	4.56 ± 0.26	–	–	Phenols
Trisiloxane, 1,1,1,5,5,5-hexamethyl-3,3-bis[(trimethylsilyl)oxy]-	26.71	2.12 ± 0.04	–	–	–	–	Siloxanes
2,6,6-trimethyl-1,3-Cyclohexadiene-1-carboxaldehyde,	16.70	1.96 ± 0.16	–	–	–	–	Aldehydes
6-Amino-2,4-dimethylphenol	16.11	2.76 ± 0.17	–	–	–	–	Phenols
Benzenamine, 4-methoxy-2-methyl-	13.43	2.18 ± 0.13	–	–	–	–	Nitrogenous
1-ethyl-1H-Pyrrole-2-carboxaldehyde	10.84	4.24 ± 0.14	–	–	–	–	Aldehydes
Benzeneacetaldehyde	10.63	0.91 ± 0.05	–	–	–	–	Aldehydes
Ethyl 2-(5-methyl-5-vinyltetrahydrofuran-2-yl)propan-2-yl carbonate	12.24	–	5.00 ± 0.47	–	7.02 ± 0.51	–	Lactones
L-.alpha.-Terpineol	16.35	–	6.44 ± 0.70	–	–	–	Alcohols
2-Methoxy-6-methylaniline	13.46	–	–	2.41 ± 0.21	–	–	Nitrogenous
2,5-Dimethyl-1-propylpyrrole	16.12	–	–	2.14 ± 0.01	–	–	Nitrogenous
Citral	19.29	–	–	1.58 ± 0.12	–	–	Aldehydes
4-Hexen-1-ol, 5-methyl-2-(1-methylethenyl)-	19.86	–	–	12.7 ± 0.32	–	–	Alcohols
2-Propenamide, N-(4-methoxyphenyl)-	10.82	–	–	1.14 ± 0.02	–	–	Nitrogenous
Indolizine	20.30	–	–	5.39 ± 0.22	–	–	Heterocycles
N,N-dimethyl-2-Pyrazinamine	10.82	–	–	–	7.71 ± 0.50	–	Nitrogenous
Benzenamine, 2-methoxy-4-methyl-	13.44	–	–	–	3.23 ± 0.26	–	Nitrogenous
5-Ethoxy-2-oxiran-2-yl-pyridine	16.10	–	–	–	5.14 ± 0.51	–	Nitrogenous
Methyl salicylate	16.51	–	–	–	6.78 ± 0.49	–	Lactones
Decanal	16.95	–	–	–	2.59 ± 0.46	–	Aldehydes
8-methyl-8-Azabicyclo[3.2.1]oct-2-ene	10.83	–	–	–	–	2.61 ± 0.07	Nitrogenous
Bicyclo[3.1.1]heptan-3-one, 2-hydroxy-2,6,6-trimethyl-	26.54	–	–	–	–	13.1 ± 0.10	Ketones
2(4H)-Benzofuranone, 5,6,7,7a-tetrahydro-4,4,7a-trimethyl-	27.59	–	–	–	–	1.95 ± 0.03	Lactones

*RT* Retention time (min), *ZY* Zhuye, *TF* Tuofu, *JHX* Jianghuaxiang, *JD* Juduo, *YSX* Yashixiang.

### Hierarchical cluster analysis of aromatic composition of 5 PDC teas

**Figure 3:**
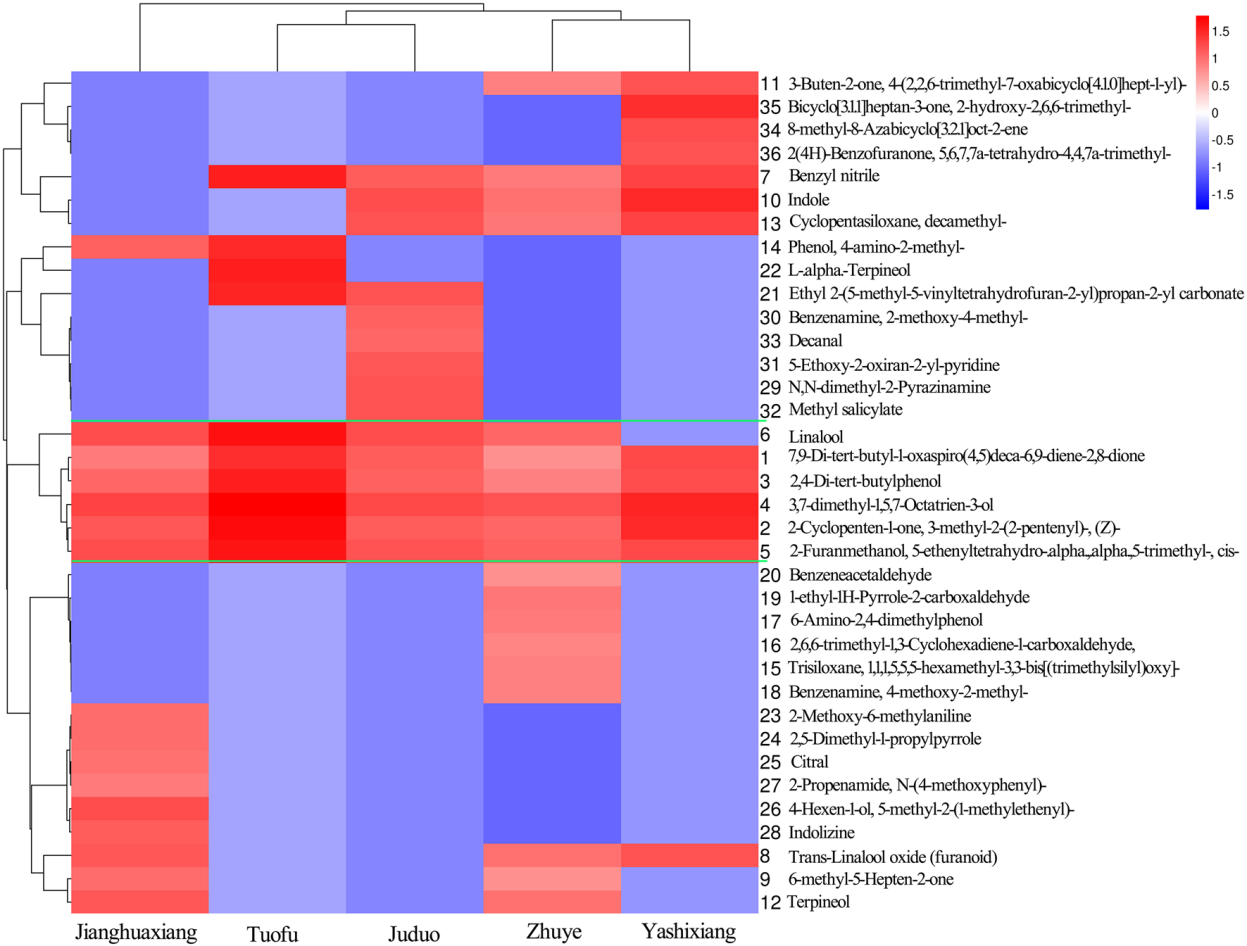
Heat map of hierarchical clustering of different aromatic components of 5 kinds of these 4 aroma types PDC tea samples.

Hierarchical cluster analysis, which is mostly used to analyze multivariate data, is a multivariate statistical method that gradually aggregates samples based on the similarity or proximity of their quality features. Currently, it is widely used to distinguish tea varieties and identify tea quality^22^. For example, Wang^23^ used hierarchical cluster analysis to distinguish black teas from 7 districts in Guangdong Province; Liu^6^ characterized the flavor profiles of 20 compounds in 16 green tea samples from different geographic origins; Wang^24^ used hierarchical cluster analysis to clearly distinguish two types of oolong tea based on their colors. In this study, the distribution of the 36 aromatic components in 5 PDC teas was also analyzed using hierarchical cluster analysis. The heat map clearly shows that these 40 aromatic components are mainly divided into 3 categories (Fig. 3). The first category is the Linalool, the Benzyl nitrate, the 7,9-Di-tert-butyl-1-oxaspiro(4,5)deca-6,9-diene-2,8-dione, the 2,4-Di-tert-butylphenol, the 3,7-dimethyl-1,5,7-Octatrien-3-ol, the 2-Cyclopenten-1-one, and the 2-Furanethanol,5-ethyltetrahydro-.alpha.,.alpha.,5-trimethyl-,cis- composition, most of which are ketones and alcohols, which are almost all aromatic components of these 5 kinds tea. The second category is the 3-Buten-2-one,4-(2,2,6-trimethyl-7-oxabicyclo[4.1.0]hept-1-yl), the Bicyclo[3.1.1]heptan-3-one,2-hydroxy-2,6,6-trimethyl-, the 8-methyl-8-Azabicyclo[3.2.1]oct-2-ene, the 2(4H)-Benzofuranone,5,6,7,7a-tetrahydro-4,4,7a-trimethyl-, the Benzyl nitrile, the Indole, the Cyclopentasiloxane, decamethyl-, the Phenol, 4-amino-2-methyl-, the L-.alpha.-Terpineol, the Ethyl2-(5-methyl-5-vinyltetrahydrofuran-2-yl)propan-2-yl carbonate, the Benzenamine,2-methoxy-4-methyl-, the Decanal, the 5-Ethoxy-2-oxiran-2-yl-pyridine and the N,N-dimethyl-2- Pyrazinamine, the Methyl salicylate. These aromatic components differed comparatively among these 5 kinds of PDC tea, with the greatest differences being in the remaining 15 aromatic components of the third category. Differences in the types and relative contents of aroma components are the material basis for the differences in the quality of aroma. Combined with the hierarchical clustering analysis, the 1st category is containing the aroma components common to these 5 kinds of PDC teas, which may then be the aroma components that most PDC tea varieties have, retaining the aromatic traits of the parent plant before improvement. The most significant differences in aroma components among these 5 kinds of PDC teas are clustered in the 2nd and 3rd categories, indicating that the improvement of the variety has led to changes in the relative content and composition of internal substances in the tea, which ultimately result in differences in the taste of the tea soup. Therefore, this study can confirm that the differences in the aroma composition of these 5 kinds of PDC tea are all concentrated in the 2nd and 3rd categories, which may also be the root cause of the differences in these teas. Of course, in order to comprehensively analyze the differences between these teas, we have included in the subsequent discussion and analysis a discussion of the relevance of the results to other analyses.

**Figure 4:**
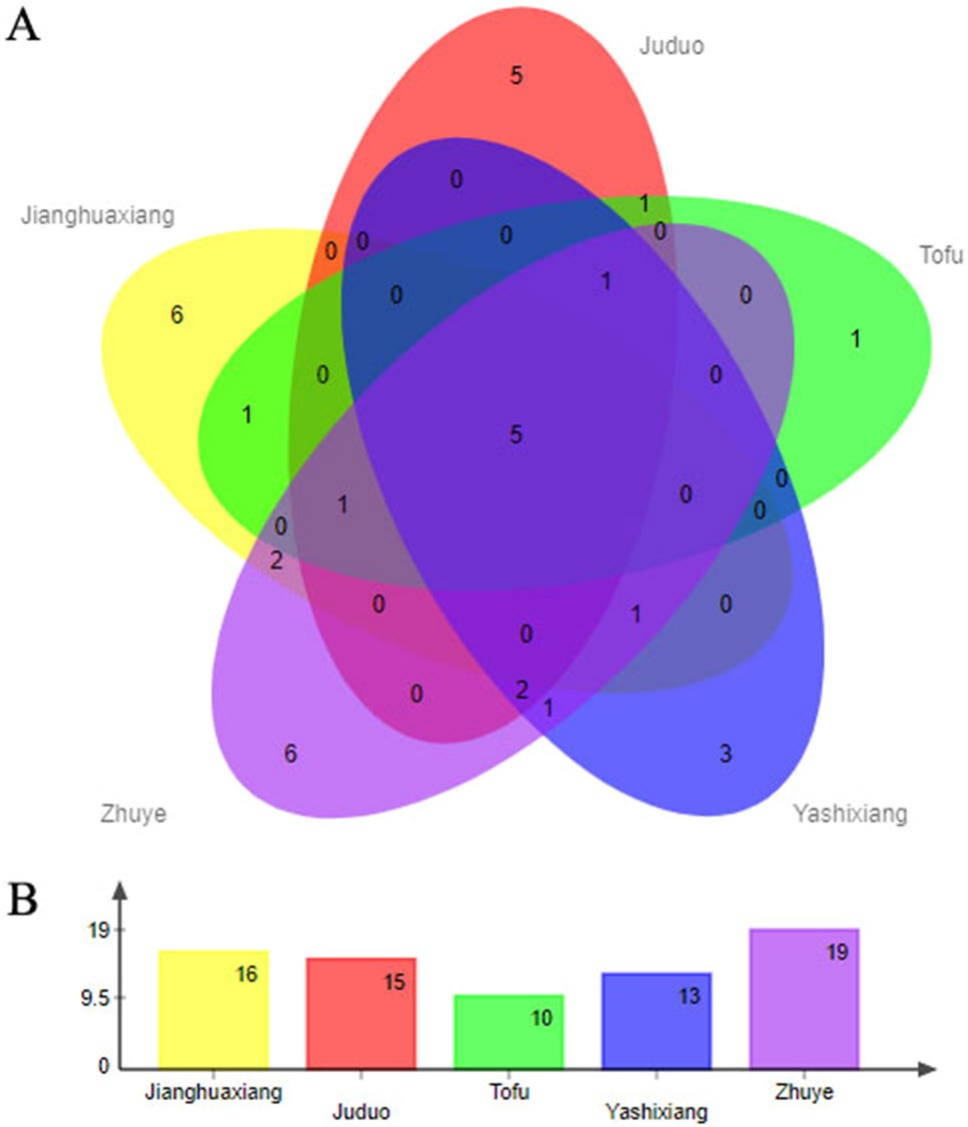
Wayne plots (**A**) and number of aroma component species (**B**) of aroma component species of 5 kinds of these 4 aroma types PDC tea samples.

In addition, combining the results of Fig. 3 and Table 1, it can be found that the 7,9-Di-tert-butyl-1-oxaspiro(4,5)deca-6,9-diene-2,8-dione, the 2-Cyclopenten-1-one,3-methyl-2-(2-pentenyl)-,(Z)-, the 2,4-Di-tert-butylphenol, the 3,7-dimethyl-1,5,7-Octatrien-3-ol and the 2-Furanmethanol,5-ethenyltetrahydro-.alpha.,.alpha.,5-trimethyl-,cis- these aromatic components are these 5 kinds tea shared, and the relative content of the 3,7-dimethyl-1,5,7-Octatrien-3-ol is the highest among these 5 kinds tea. Comparing these aromatic components, it was found that the 2-Methoxy-6-methylaniline, the 2,5-Dimethyl-1-propelpyrole, the Citral, the 4-Hexen-1-ol,5-methyl-2-(1-methylethynyl)-, the 2-Propenamide,N-(4-methoxyphenyl)-, and the Indizine are unique to Jianghuaxiang; The Trisiloxane, the 1,1,1,5,5,5-hexamethyl-3,3-bis[(trimethylsilyl)oxy]-, the 2,6,6-trimethyl-1,3-Cyclohexadiene-1-carboxaldehyde, the 6-Amino-2,4-dimethylphenol, the Benzenamine,4-methoxy-2-methyl-, the 1-ethyl-1H-Pyrrole-2-carboxaldehyde and the Benzeneacetaldehyde are unique to Zhuye; The 8-methyl-8-Azabicyclo[3.2.1]oct-2-ene, the Bicyclo[3.1.1]heptan-3-one, the 2-hydroxy-2,6,6-trimethyl- and the 2(4H)-Benzofuranone,5,6,7,7a-tetrahydro-4,4,7a-trimethyl- are unique to Yashixiang; The L-.alpha.-Terpineol are unique to Tuofu; The N,N-dimethyl-2-Pyrazinamine, the Ethyl2-(5-methyl-5-vinyltetrahydrofuran-2-yl)propan-2-yl carbonate, the Benzenamine,2-methoxy-4-methyl-, the 5-Ethoxy-2-oxiran-2-yl-pyridine, the Methyl salicylate and the Decanal are unique to Juduo. Zhuye and Jianghuaxiang has a maximum of 6 unique aromatic components, followed by Juduo with 5, and Yashixiang and Tuofu with 3 and 1, respectively (Fig. 4A). These differences in the types and relative contents of aromatic components may be the material basis for the differences in aroma quality and taste of these 5 PDC teas. In addition, clustering based on different tea reveals that the similarity ranking sequences are Juduo, Tuofu, Yashixiang, Zhuye, and Jianghuaxiang. Surprisingly, both Tuofu and Yashixiang belong to the Xingrenxiang tea type, which means that we can use similar methods to distinguish and identify which type of aroma tea different varieties of PDC tea belong to. Of course, more evidence is needed to fully evaluate and justify this.

### Combination differences and composition regularity of aromatic components of 5 PDC teas

**Figure 5:**
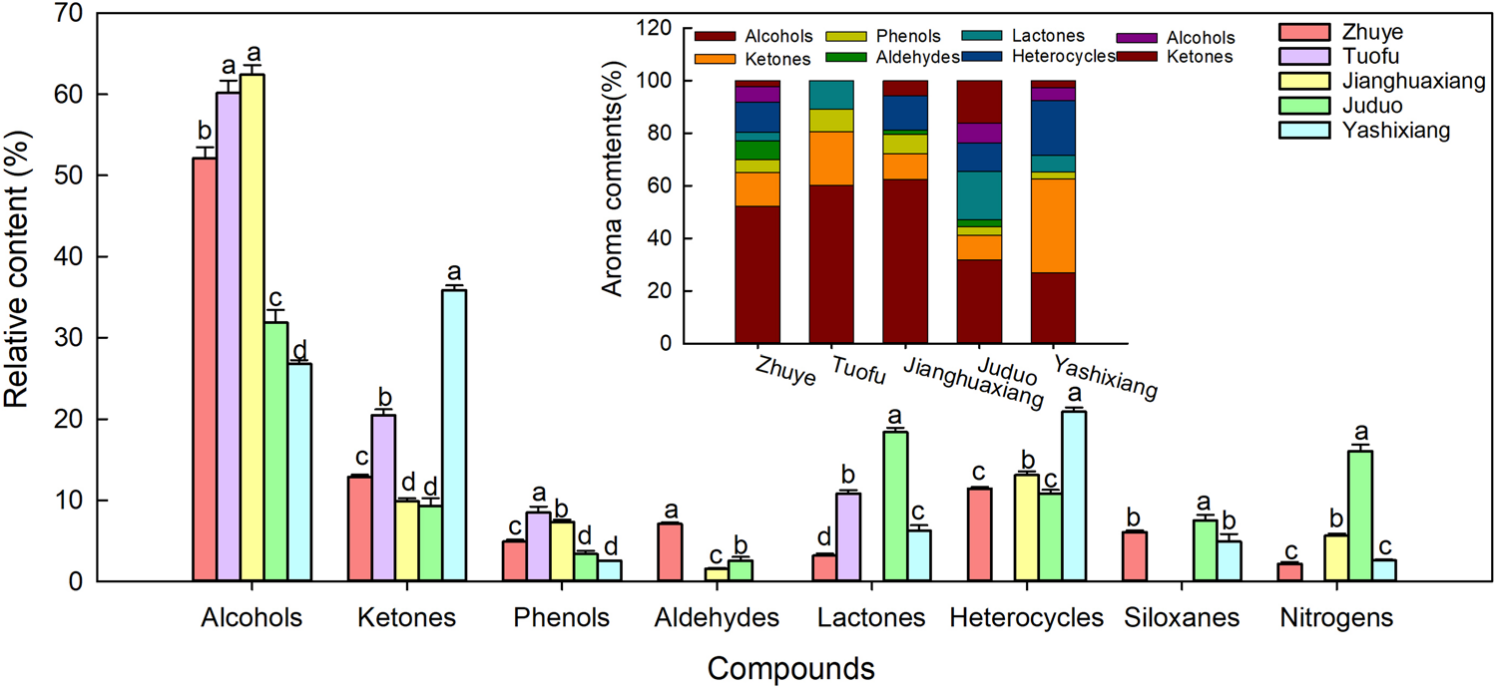
Comparison among volatile compounds in 5 kinds of these 4 aroma types PDC tea samples. All data are presented as mean ± standard error (SE, n = 3). Different letters above bars indicate statistical significance (*P* < 0.05).

After classifying the 36 identified aromatic components, Tuofu was found to have the least variety of aromatic components, followed by Yashixiang and Juduo, the highest is Zhuye (Table 1). The relative content of alcohols in Zhuye was the highest (52.13%), followed by ketones (12.90%) and heterocycles (11.45%), and lowest for nitrogenous (2.18%). The relative content of alcohols in Tuofu was the highest (60.16%), followed by ketones (20.47%), then lactones (10.86%) and phenols (8.51%). The relative content of alcohols in Jianghuaxiang was the highest (62.41%), followed by heterocycles (13.11%) and ketones (9.88%) and the lowest was aldehydes (1.58%). The relative content of alcohols in Juduo was the highest (31.88%) followed by lactones (18.41%) and nitrogens (16.08%) and the least was aldehydes (2.58%). The relative content of ketones in Yashixiang was the highest (35.87%), followed by alcohols (26.84%) and heterocycles (20.93%), and the least were phenols (2.58%) and nitrogens (2.62%). In addition, this study also found that alcohols, ketones and phenols were common to these 5 PDC teas, and alcohols accounted for the largest relative content, followed by ketones, and the smallest was phenols (Table 2 and Fig. 5), which suggests that in terms of the relative content of alcohols and ketones dominated the aroma substances of PDC tea. Alcohols identified in the 5 PDC teas, Zhuye, Tuofu, Jianghuaxiang, Juduo, and Yashixiang, accounted for 52.13%, 60.16%, 62.41%, 31.88%, and 26.84% of their total content, respectively. Alcohols is an important aromatic-active compounds. Studies have shown that linalool, benzyl alcohol and geraniol are the main alcohol in green tea^6,25^. Unlike green tea, the main alcohol in this study is the 3,7-dimethyl-1,5,7-octatrien-3-ol (27.7%, 30.6%, 19.9%, 14.8% and 22.8%), and of course, linalool also played a role. In addition, the relative content of alcohol in Zhuye, Tuofu and Jianghuaxiang is significantly higher than Juduo and Yashixiang, which means that the aromatic odor provided by alcohol is the key source and typical aroma characteristics of the aroma of Zhuye, Tuofu and Jianghuaxiang, while 3,7-dimethyl-1,5,7-octatrien-3-ol is their core aroma substance.

**Table 2:** Relative contents of various aromatic components of 5 kinds of these 4 aroma types PDC tea samples.

Category	Relative content (%)				
	Zhuye	Tuofu	Jianghuaxiang	Juduo	Yashixiang
Alcohols	52.13 ± 1.32	60.16 ± 1.53	62.41 ± 1.20	31.88 ± 1.55	26.84 ± 0.43
Ketones	12.90 ± 0.25	20.47 ± 0.74	9.88 ± 0.38	9.30 ± 0.97	35.87 ± 0.59
Phenols	4.95 ± 0.21	8.51 ± 0.72	7.34 ± 0.27	3.39 ± 0.42	2.58 ± 0.05
Aldehydes	7.11 ± 0.20	–	1.58 ± 0.12	2.58 ± 0.46	–
Lactones	3.19 ± 0.26	10.86 ± 0.40	–	18.41 ± 0.55	6.25 ± 0.72
Heterocycles	11.45 ± 0.21	–	13.11 ± 0.48	10.83 ± 0.45	20.93 ± 0.50
Siloxanes	6.09 ± 0.21	–	–	7.52 ± 0.68	4.91 ± 0.94
Nitrogens	2.18 ± 0.23	–	5.68 ± 0.23	16.08 ± 0.81	2.62 ± 0.12

In addition, ketones were another type key aromatic active compound in the 5 PDC teas. Ketones identified in Zhuye, Tuofu, Jianghuaxiang, Juduo and Yashixiang accounted for 12.90%, 20.47%, 9.88%, 9.30% and 35.87% of the total, respectively. One of the shared ketones substances is 2-Cyclopenten-1-one,3-methyl-2-(2-pentenyl)-,(Z)-, which is the most abundant, and thus may be an important contributor to the flavor of these 5 PDC teas. Some studies have demonstrated that ketones contribute significantly to the aroma of tea because of their low overall odor threshold and high flavor dilution coefficient^6,26^. Ketones substance formation may be related to the degradation of unsaturated fatty acids and β-carotene within tea^27,28^.

In addition to abundant alcohol and ketones, phenols, aldehydes, lactones, heterocycles, siloxanes and nitrogenous also exist in these 5 PDC teas. It has been confirmed that phenols determine the bitter and astringent taste of tea^29^, and although the relative content of phenols is not high, they are present in all 5 PDC teas, implying that it is also a relatively important contributor to the flavor of PDC tea. The production of aldehydes substances in tea is mainly associated with the degradation of unsaturated fatty acids^30,31^. Aldehydes are considered to play an important role in the formation of aroma due to their extremely low odor threshold and similar odor characteristics^29,32^. In addition, lactones, heterocycles, siloxanes and nitrogenous are important aromatic components in tea. Lactones may give tea fruit flavor. Some heterocycles may be key substances in the formation of tea aroma. For example, indole has sweet taste and floral aroma, which can effectively improve the overall aroma of tea^33^. Siloxanes is also a key aroma component in tea, which appears to increase and then decrease during the preparation of tea samples^34^. Nitrogenous has a special nutty flavor, and its formation may be related to the production of amino acids and sugars^35,36^. However, this study found that aldehydes only existed in Zhuye, Jianghuaxiang and Juduo, while lactones did not exist in Jianghuaxiang. Heterocycles and nitrogenous are absent in Tuofu. Siloxanes only exist in Zhuye, Juduo and Yashixiang. Therefore, it is speculated that the distribution differences of these aroma types in 5 kinds of PDC tea may be the key factor of their flavor differences. It needs to be considered that our study did not use internal standards for quantitative analysis. Although we only wanted to explore the combination of aroma components, composition patterns, and core aroma substances of these four aroma types and five *Phoenix Dancong* teas. To be sure it is very important to quantify with internal standards, can we get similar conclusions if we have used internal standards for quantitative analysis? So, if we want to establish a complete method for identifying the varieties of *Phoenix Dancong* tea through differences in different aroma components, more experimental evidence is needed to prove and improve in the future.

### PCA of aromatic components of 5 PDC teas

**Figure 6:**
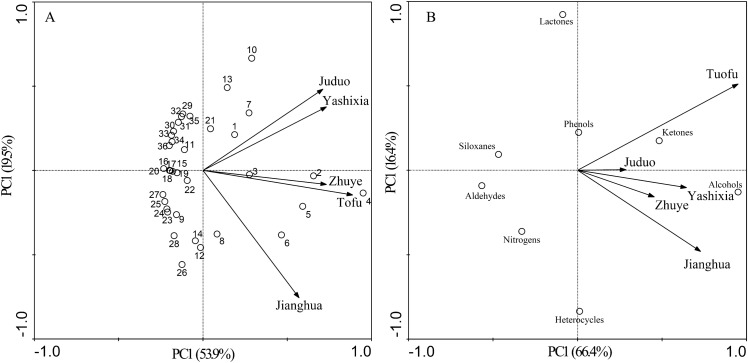
Principal component analysis of each aroma component (**A**) and each type of aroma component (**B**) of five PDC tea samples. **1:** 7,9-Di-tert-butyl-1-oxaspiro(4,5)deca-6,9-diene-2,8-dione; **2:** 2-Cyclopenten-1-one, 3-methyl-2-(2-pentenyl)-, (Z)-; **3:** 2,4-Di-tert-butylphenol; **4:** 3,7-dimethyl-1,5,7-Octatrien-3-ol; **5:** 2-Furanmethanol, 5-ethenyltetrahydro-.alpha.,.alpha.,5-trimethyl-, cis-; **6:** Linalool; **7:** Benzyl nitrile; **8:** trans-Linalool oxide (furanoid); **9:** 6-methyl-5-Hepten-2-one; **10:** Indole; **11:** 3-Buten-2-one, 4-(2,2,6-trimethyl-7-oxabicyclo[4.1.0]hept-1-yl)-; **12:** Terpineol; **13:** Cyclopentasiloxane, decamethyl-; **14:** Phenol, 4-amino-2-methyl-; **15:**Trisiloxane, 1,1,1,5,5,5-hexamethyl-3,3-bis[(trimethylsilyl)oxy]-; **16:** 2,6,6-trimethyl-1,3-Cyclohexadiene-1-carboxaldehyde,; **17:** 6-Amino-2,4-dimethylphenol; **18:** Benzenamine, 4-methoxy-2-methyl-; **19:** 1-ethyl-1H-Pyrrole-2-carboxaldehyde; **20:** Benzeneacetaldehyde; **21:** Ethyl 2-(5-methyl-5-vinyltetrahydrofuran-2-yl)propan-2-yl carbonate; **22:** L-.alpha.-Terpineol; **23:** 2-Methoxy-6-methylaniline; **24:** 2,5-Dimethyl-1-propylpyrrole; **25:** Citral; **26:** 4-Hexen-1-ol, 5-methyl-2-(1-methylethenyl)-; **27:** 2-Propenamide, N-(4-methoxyphenyl)-; **28:** Indolizine; **29:** N,N-dimethyl-2-Pyrazinamine; **30:** Benzenamine, 2-methoxy-4-methyl-; **31:** 5-Ethoxy-2-oxiran-2-yl-pyridine; **32:** Methyl salicylate; **33:** Decanal; **34:** 8-methyl-8-Azabicyclo[3.2.1]oct-2-ene; **35:** Bicyclo[3.1.1]heptan-3-one, 2-hydroxy-2,6,6-trimethyl-; **36:** 2(4H)-Benzofuranone, 5,6,7,7a-tetrahydro-4,4,7a-trimethyl-.

PCA is a non-target statistical method to categorize composite indicators according to a certain regular, which can highlight the similarities and differences between samples and sample content, and provide the objects contributing key components^6^. Studies have confirmed that PCA can reveal the similarities and differences between different teas according to the types and contents of aromatic components in tea^37,38^. PCA was used in this study to reflect the contribution of each variable to the 5 PDC teas. The first principal component (PC1) explained 53.9% of the variation and the second principal component (PC2) explained 19.5% of the variation of various aromatic components in the refined finished tea of 5 PDC teas (Fig. 6A). It is mainly contributed by the 3,7-dimethyl-1,5,7-Octatrien-3-ol, the 2-Cyclopenten-1-one, 3-methyl-2-(2-pentenyl)-, (z)-, the 2-furanmethanol,5-ethyltetrahydro-.alpha.,.alpha.,5-trimethyl-,cis-, the linalool, the indole and the Cyclopentasiloxane, decamethyl-. The first principal component (PC1) explained 66.4% of the variation and the second principal component (PC2) explained 16.4% of the variation in all types of aromatic components in the refined finished tea of 5 PDC teas (Fig. 6B), with the first principal component (PC1) being mainly contributed by alcohols and ketones. Our study showed that the aroma characteristics of 5 PDC teas were significantly different, but alcohols (the 3,7-dimethyl-1,5,7-octatrien-3-ol, the 2-furanmethanol,5-ethyltetrahydro-.alpha.,.alpha.,5-trimethyl-,cis- and the Linalool) and ketones (the 2-cyclopenten-1-one, the 3-methyl-2-(2-pentenyl)-,(z)-) may be the core aromatic components, and the differences in flavor in the teas were mainly due to differences in the combination and content of aromatic components such as alcohols, ketones, lactones, heterocycles, siloxanes and nitrogenous.

## Materials and methods

### Experimental materials

This study was conducted at Hanshan Normal University, Chaozhou City, Guangdong Province, China. Five different varieties of four aroma types PDC tea, Zhuye, Tuofu, Jianghuaxiang, Juduo, Yashixiang were used as the research objects. In the spring of 2022, fresh leaves of PDC tea with an average age of 10 years were taken from Fenghuang Mountain in the north of Fenghuang Town, Chaoan District, Chaozhou City, Guangdong Province using a multi-point sampling method. Fenghuang Mountain is located approximately 30 km from the urban area. The entire mountain is steep and upright, with its main peak at an altitude of 1497.8 m. The climate of this region belongs to the subtropical climate zone, with abundant sunshine and rainfall. The average temperature is about 21.4 °C, with the highest temperature recorded at 39.6 °C and the lowest temperature recorded at − 0.5 °C. The average annual rainfall is 1685.8 mm. It has fertile, acidic soil with abundant sunlight resources, which is extremely suitable for the growth of certain varieties of tea trees. Liu et al.^39^ have described the source of PDC tea and the identification of different varieties. In addition, the PDC tea specimen information is not yet public and needs to be kept confidential, it is temporarily stored in the herbarium of Hanshan Normal University. During sample collection, the tea leaves harvested from each tea tree are used as one replicate, and each tea tree variety undergoes three replicates, all tea tree variety come from the same tea garden.

### Sample preparation and HS-SPME extraction of tea aromatic components

Fresh leaves of 5 different varieties of 4 aroma types PDC tea were selected to be made into tea according to the unified processing technology of local PDC tea^2^, and the finished tea after refining was taken as the test sample (Fig. 1). The SPME stable flex fiber (DVB/CAR/PDMS, 50/30 μm) from Supelco (Oakville, Canada) was used for the headspace experiments. SPME fiber length 1 cm. Before sample extraction, the extraction head was aged at 250 °C for 1 h in the GC injection port. Weigh 4 g of refined finished tea and add it to a beaker filled with 200 mL of boiling water to brew tea soup (Fig. 1). Then use a pipette to transfer 5 mL of brewed tea soup into a 20 mL headspace bottle, immediately seal the bottle mouth, equilibrium time for 10 min, and maintain an equilibrium temperature of 70 °C. Perform SPME adsorption extraction for 30 min, place the fibers in the GC syringe port, and perform thermal desorption at 250 °C for 5 min, after which the tea aromatic components of each tea were detected by GC–MS.

### GC–MS analysis of aroma composition of tea leaves

In this study, an Agilent 7890B gas chromatograph equipped with a 5977B mass spectrometer was used to analyze and determine of aromatic components of Zhuye, Tuofu, Jianghuaxiang, Juduo, and Yashixiang. Chromatographic conditions: chromatographic column was an DB-5MS capillary column (30 m × 0.25 m, 0.25 μm film thickness), sample inlet temperature 250 °C, using high-purity helium as the gas carrier (purity > 99.99%), the gas flow rate of 1 mL/min; injection volume of 2 µL, and a non-split injection mode is used. The GC oven temperature program is as follows: from 40 °C (maintained for 5 min), increase to 230 °C at 5 °C/min, and then increase to 250 °C at 15 °C/min; the solvent was delayed time for 3 min. Mass spectrometry conditions: transfer line temperature of 280 °C, ion source temperature of 230 °C, quadrupole temperature of 150 °C, electron energy of 70 eV; The scan range was m/z 30–400 and the solvent delay time was 0 min. The mass spectrometry data obtained from GC–MS conditions were searched in the data library of the National Institute of Standards and Technology (NIST98 MS), the components with matching degree greater than 70 were retained, and the relevant mass spectral data were checked to analyze the base fronts, the core-to-mass ratios and the relative abundances, etc., and the peaks were confirmed. Volatile substances that play an important role in the formation of aroma quality in PDC tea were further identified through standard samples. Retention indices of the compounds were calculated using an n-alkane (C8–C32) (Linalool (≥ 98%), L-.alpha.-Terpineol (≥ 96%), Citra (≥ 98%), Indole (> 99.5%), Methyl salicylate (≥ 99%), Benzeneacetaldehyde (≥ 95%), Decanal (≥ 98.2%) etc.) series (Sigma-Aldrich, USA), and comparative identification of retention indices in the literature as well as in databases^14,40^. The peak area normalization method is used to calculate the content of each component, and the relative content of each aroma component is represented by the ratio of its peak area to the total peak area^41^. Three biological replications were performed for each tea variety.

### Statistical analysis

The preliminary processing of the data was completed by Excel 2016, then statistic tests were performed in SPSS 18 (SPSS Inc., Chicago, IL, USA) software, and followed by the use of Sigmaplot14 (Systat Software, San Jose, CA, USA) software to analyze the differences in aromatic components of 5 kinds tea. The hierarchical clustering heat map and Wayne map of the different aromatic components of 5 kinds tea were plotted using R software. Finally, the PCA of each aroma component and various volatile compounds of 5 kinds tea was conducted using Canoco 4.5 (Microcomputer Power, USA).

### Statement

All materials in the manuscript were collected with permission from the Chaozhou Weiye Tea Garden. The collection and experiments on plant materials for this study complies with the IUCN Policy Statement on Research Involving Species at Risk of Extinction and the Convention on the Trade in Endangered Species of Wild Fauna and Flora.

## Conclusions

In this study, a total of 40 major aromatic components were detected based on GC–MS of 5 kinds of these 4 aroma types PDC tea, including Zhuye, Tuofu, Jianghuaxiang, Juduo and Yashixiang identified 21, 10, 17, 16 and 14 aromatic components, respectively. And the 7,9-Di-tert-butyl-1-oxaspiro(4,5)deca-6,9-diene-2,8-dione, the 2-Cyclopenten-1-one,3-methyl-2-(2-pentenyl)-,(Z)-, the 2,4-Di-tert-butylphenol, the 3,7-dimethyl-1,5,7-Octatrien-3-ol and 2-Furanmethanol,5-ethenyltetrahydro-.alpha.,.alpha.,5-trimethyl-,cis- these aromatic components are common to these 5 kinds teas. The results of the PCA showed that alcohols and ketones were the main types of aromatic components of these 5 kinds tea, with a high contribution to the aroma of the tea, indicating that the composition and content of the aromatic components of these 5 PDC teas had a certain regularity, which included the differences in the content of the common components as well as the differences in the types of the unique aromatic components. Combined with the clustering heat map analysis, the similarity ranking sequence was Juduo, Tuofu, Yashixiang, Zhuye and Jianghuaxiang. These new findings clarified the differences of aromatic components, main types and similarity of aromatic components of the 5 kinds tea. The continuous development of tea cultivation technology, so that in the same aroma tea and evolved a variety of varieties of tea. However, the current assessment of different types of PDC tea varieties remain in the subjective awareness of the determination, the lack of scientific and effective uniform standards. This will lead to incorrect evaluation of a certain variety of tea as belonging to that type of aroma tea. In the future, it may be possible to use this study as a basis to form identification standards to distinguish and identify the new variety of PDC tea belongs to which type aroma types tea using similar methods. In addition, with the help of relevant analytical methods, this study clustered the aromatic components of various aroma types of PDC tea to reveal the core aroma-endowed substances as well as the regularity of the combination and content of aromatic components among different aroma types of PDC tea, which can provide theoretical foundation and scientific basis for the development of PDC tea, breeding and study of the relationship between the evolution of varieties.

### Supplementary Information

Supplementary Table S1.

## Data Availability

All data generated or analysed during this study are included in this published article.

## References

[1] Li Z (2022). Comparative analysis of Fenghuang Dancong, Tieguanyin, and Dahongpao teas using headspace solid-phase microextraction coupled with gas chromatography-mass spectrometry and chemometric methods. PLoS ONE.

[2] Li Z, Wang J (2020). Identification and similarity analysis of aroma substances in main types of Fenghuang Dancong tea. PLoS ONE.

[3] Xu M, Wang J, Zhu LY (2020). Tea quality evaluation by applying E-nose combined with chemometrics methods. Int. J. Food Sci. Tech..

[4] Zhao J, Liu W, Chen Y (2022). Identification of markers for tea authenticity assessment: Non-targeted metabolomics of highly similar oolong tea cultivars (*Camellia sinensis* var. sinensis). Food Control.

[5] Yang YQ, Yin HX, Yuan HB (2018). Characterization of the volatile components in green tea by IRAE-HS-SPME/GC-MS combined with multivariate analysis. PLoS ONE.

[6] Liu P, Zheng P, Gong Z (2020). Comparing characteristic aroma components of bead-shaped green teas from different regions using headspace solid-phase microextraction and gas chromatography–mass spectrometry/olfactometry combined with chemometrics. Eur. Food Res. Technol..

[7] Chen X, Chen D, Jiang H (2018). Aroma characterization of Han-zhong black tea (*Camellia sinensis*) using solid phase extraction coupled with gas chromatography-mass spectrometry and olfactometry and sensory analysis. Food Chem..

[8] Zhu Y, Lv H, Shao C (2018). Identification of key odorants re-sponsible for chestnut-like aroma quality of green teas. Food Res. Int..

[9] Shi J, Zhu Y, Zhang Y, Lin Z, Lv HP (2019). Volatile composition of Fu-brick tea and Pu-erh tea analyzed by comprehensive two-dimensional gas chromatography-time-of-flight mass spectrometry. LWT Food Sci. Technol..

[10] Shi Y, Wang M, Dong Z (2021). Volatile components and key odorants of Chinese yellow tea (*Camellia sinensis*). LWT Food Sci. Technol..

[11] Xu YQ, Liu PP, Shi J (2018). Quality development and main chemical components of Tieguanyin oolong teas processed from different parts of fresh shoots. Food Chem..

[12] He C, Li Y, Zhou J (2022). Study on the suitability of tea cultivars for processing oolong tea from the perspective of aroma based on olfactory sensory, electronic nose, and GC-MS data correlation analysis. Foods.

[13] Yang Z, Baldermann S, Watanabe N (2013). Recent studies of the volatile compounds in tea. Food Res. Int..

[14] Liu PP, Yin JF, Chen GS, Wang F, Xu YQ (2018). Flavor characteristics and chemical compositions of oolong tea processed using different semi-fermentation times. J. Food Sci. Technol..

[15] He C, Li Z, Liu H, Zhang H, Wang L, Chen H (2020). Characterization of the key aroma compounds in *Semnostachya menglaensis* Tsui by gas chromatography-olfactometry, odor activity values, aroma recombination, and omission analysis. Food Res. Int..

[16] Shevchuk A, Jayasinghe L, Kuhnert N (2018). Differentiation of black tea infusions according to origin, processing and botanical varieties using multivariate statistical analysis of LC-MS data. Food Res. Int..

[17] Belmonte-Sánchez JR, Romero-González R, Martínez VJL, Arrebola FJ, Garrido FA (2020). 1H NMR and multi-technique data fusion as metabolomic tool for the classification of golden rums by multivariate statistical analysis. Food Chem..

[18] Jing J, Shi Y, Zhang Q, Wang J, Ruan J (2017). Prediction of Chinese green tea ranking by metabolite profiling using ultra-performance liquid chromatography–quadrupole time of flight mass spectrometry (UPLC–Q-TOF/MS). Food Chem..

[19] Meng W, Xu X, Cheng KK (2017). Geographical origin discrimination of oolong tea (TieGuanYin, *Camellia sinensis* (L.) O. Kuntze) using proton nuclear magnetic resonance spectroscopy and near-infrared spectroscopy. Food Anal. Methods.

[20] Yang Y, Zhang M, Yin H (2018). Rapid profiling of volatile compounds in green teas using micro-chamber/thermal extractor combined with thermal desorption coupled to gas chromatographymass spectrometry followed by multivariate statistical analysis. LWT..

[21] Xiao L, Mao S, Tong H (2018). Analysis of volatile components of three types of Fenghuang Dancong tea. Food Sci..

[22] Chen Q, Zhang M, Chen M, Li M, Gao X (2021). Influence of *Eurotium cristatum* and *Aspergillus niger* individual and collaborative inoculation on volatile profile in liquid-state fermentation of instant dark teas. Food Chem..

[23] Wang Q, Qin D, Jiang X, Fang K, Li B, Wang Q (2023). Characterization of the aroma profiles of Guangdong black teas using non-targeted metabolomics. Foods.

[24] Wang C, Lv S, Wu Y, Gao X, Li J, Zhang W (2016). Oolong tea made from tea plants from different locations in Yunnan and Fujian, China showed similar aroma but different taste characteristics. Springerplus.

[25] Wu Y, Lv S, Lian M, Wang C, Gao X, Meng Q (2016). Study of characteristic aroma components of baked Wujiatai green tea by HS-SPME/GC-MS combined with principal component analysis. CyTA-J. Food.

[26] Wu Y, Lv S, Wang C, Gao X, Li J, Meng Q (2016). Comparative analysis of volatiles difference of Yunnan sun-dried pu-erh green tea from different tea mountains: Jingmai and Wuliang mountain by chemical fingerprint similarity combined with principal component analysis and cluster analysis. Chem. Cent. J..

[27] Ravichandran R, Parthiban R (2000). Lipid occurrence, distribution and degradation to flavour volatiles during tea processing. Food Chem..

[28] Ravichandran R (2002). Carotenoid composition, distribution and degradation to flavour volatiles during black tea manufacture and the effect of carotenoid supplementation on tea quality and aroma. Food Chem..

[29] Liu X, Liu Y, Li P (2021). Chemical characterization of Wuyi rock tea with different roasting degrees and their discrimination based on volatile profiles. RSC Adv..

[30] Kim Y, Lee KG, Kim MK (2016). Volatile and non-volatile compounds in green tea affected in harvesting time and their correlation to consumer preference. J. Food Sci. Technol..

[31] Kebede BT, Grauwet T, Mutsokoti L (2014). Comparing the impact of high-pressure high temperature and thermal sterilization on the volatile fingerprint of onion, potato, pumpkin and red beet. Food Res. Int..

[32] Wang Q, Jiang X, Qin D (2020). Metabolic profiling of flavor compounds in black teas with almond odor during processing. Eur. Food Res. Technol..

[33] Jumtee K, Komura H, Bamba T, Fukusaki E (2011). Predication of Japanese green tea (Sen-cha) ranking by volatile profiling using gas chromatography mass spectrometry and multivariate analysis. J. Biosci. Bioeng..

[34] Flaig M, Schieberle P (2020). Characterization of the key odorants in a high-grade chinese green tea beverage (*Camellia sinensis*; Jingshan cha) by means of the sensomics approach and elucidation of odorant changes in tea leaves caused by the tea manufacturing process. J. Agric. Food Chem..

[35] Sheibani E, Duncan SE, Kuhn DD (2016). Changes in flavor volatile composition of oolong tea after panning during tea processing. Food Sci. Nutr..

[36] Eric K, Raymond LV, Huang M (2013). Sensory attributes and antioxidant capacity of Maillard reaction products derived from xylose, cysteine and sunflower protein hydrolysate model system. Food Res. Int..

[37] Xu YQ, Wang C, Li CW (2016). Characterization of aroma-active compounds of puerh tea by headspace solid-phase microextraction (HS-SPME) and simultaneous distillation-extraction (SDE) coupled with GColfactometry and GC-MS. Food Anal. Methods.

[38] Wang B, Chen H, Qu F (2021). Identification of aroma-active components in black teas produced by six Chinese tea cultivars in high-latitude region by GC–MS and GC–O analysis. Eur. Food Res. Technol..

[39] Liu YQ (2022). Comparative phylogenetic analysis of oolong tea (*Phoenix Dancong* tea) using complete chloroplast genome sequences. Heliyon.

[40] Guo X, Song C, Ho CT, Wan X (2018). Contribution of l-theanine to the formation of 2,5-dimethylpyrazine, a key roasted peanutty flavor in oolong tea during manufacturing processes. Food Chem..

[41] Ayseli MT, Kelebek H, Selli S (2021). Elucidation of aroma-active compounds and chlorogenic acids of Turkish coffee brewed from medium and dark roasted *Coffea arabica* beans. Food Chem..

