# Supplementary material for: Exploring aromatic components differences and composition regularity of 5 kinds of these 4 aroma types Phoenix Dancong tea based on GC–MS
Source: Sci Rep. 2024 Feb 1;14:2727. doi: 10.1038/s41598-024-53307-6 (PMC10834424; doi:10.1038/s41598-024-53307-6)
Supplement: Supplementary file 1 — Supplementary Table S1. [file 41598_2024_53307_MOESM1_ESM.docx]

Table S1. List of the separated compounds in 5 kinds of these 4 aroma types PDC tea samples detected by GC-MS.

| Component | RI | amu | CAS Number |
| --- | --- | --- | --- |
| 7,9-Di-tert-butyl-1-oxaspiro(4,5)deca-6,9-diene-2,8-dione | 1928 | 276.173 | 082304-66-3 |
| 2-Cyclopenten-1-one, 3-methyl-2-(2-pentenyl)-, (Z)- | 835 | 164.12 | 000488-10-8 |
| 2,4-Di-tert-butylphenol | 1495 | 206.167 | 000096-76-4 |
| 3,7-dimethyl-1,5,7-Octatrien-3-ol | 1108 | 152.12 | 029957-43-5 |
| 2-Furanmethanol, 5-ethenyltetrahydro-.alpha.,.alpha.,5-trimethyl-, cis- | 1420 | 170.131 | 005989-33-3 |
| Linalool | 1104 | 154.136 | 000078-70-6 |
| Benzyl nitrile | 1145 | 117.058 | 000140-29-4 |
| trans-Linalool oxide (furanoid) | 1091 | 170.131 | 034995-77-2 |
| 6-methyl-5-Hepten-2-one | 953 | 126.104 | 000110-93-0 |
| Indole | 1306 | 117.058 | 000120-72-9 |
| 3-Buten-2-one, 4-(2,2,6-trimethyl-7-oxabicyclo[4.1.0]hept-1-yl)- | 1416 | 192.151 | 014901-07-6 |
| Terpineol | 1197 | 154.136 | 1000411-59-6 |
| Cyclopentasiloxane, decamethyl- | 853 | 370.094 | 000541-02-6 |
| Phenol, 4-amino-2-methyl- | 1520 | 123.068 | 002835-96-3 |
| Trisiloxane, 1,1,1,5,5,5-hexamethyl-3,3-bis[(trimethylsilyl)oxy]- | 1472 | 384.146 | 003555-47-3 |
| 2,6,6-trimethyl-1,3-Cyclohexadiene-1-carboxaldehyde, | 1204 | 150.104 | 000116-26-7 |
| 6-Amino-2,4-dimethylphenol | 2208 | 137.084 | 041458-65-5 |
| Benzenamine, 4-methoxy-2-methyl- | 926 | 137.084 | 000102-50-1 |
| 1-ethyl-1H-Pyrrole-2-carboxaldehyde | 1631 | 123.068 | 002167-14-8 |
| Benzeneacetaldehyde | 1048 | 120.058 | 000122-78-1 |
| Ethyl 2-(5-methyl-5-vinyltetrahydrofuran-2-yl)propan-2-yl carbonate | 1395 | 242.152 | 1000373-80-3 |
| L-.alpha.-Terpineol | 1186 | 154.136 | 010482-56-1 |
| 2-Methoxy-6-methylaniline | 1338 | 137.084 | 050868-73-0 |
| 2,5-Dimethyl-1-propylpyrrole | 973 | 137.12 | 020282-39-7 |
| Citral | 1602 | 152.12 | 005392-40-5 |
| 4-Hexen-1-ol, 5-methyl-2-(1-methylethenyl)- | 1183 | 154.136 | 058461-27-1 |
| 2-Propenamide, N-(4-methoxyphenyl)- | 1602 | 123.08 | 005214-29-9 |
| Indolizine | 1307 | 117.058 | 000274-40-8 |
| N,N-dimethyl-2-Pyrazinamine | 780 | 123.08 | 005214-29-9 |
| Benzenamine, 2-methoxy-4-methyl- | 1602 | 137.084 | 039538-68-6 |
| 5-Ethoxy-2-oxiran-2-yl-pyridine | 1366 | 165.079 | 1000185-71-9 |
| Methyl salicylate | 1196 | 152.047 | 000119-36-8 |
| Decanal | 1208 | 156.151 | 000112-31-2 |
| 8-methyl-8-Azabicyclo[3.2.1]oct-2-ene | 1690 | 123.105 | 000529-18-0 |
| Bicyclo[3.1.1]heptan-3-one, 2-hydroxy-2,6,6-trimethyl- | 1003 | 168.115 | 010136-65-9 |
| 2(4H)-Benzofuranone, 5,6,7,7a-tetrahydro-4,4,7a-trimethyl- | 1525 | 180.115 | 015356-74-8 |

Note: ZY=Zhuye; TF=Tuofu; JHX=Jianghuaxiang; JD=Juduo; YSX=Yashixiang
